# Optimum ratio of dietary protein and carbohydrate that maximises lifespan is shared among related insect species

**DOI:** 10.1111/acel.14067

**Published:** 2023-12-13

**Authors:** Juliano Morimoto

**Affiliations:** ^1^ Institute of Mathematics, University of Aberdeen, King's College Aberdeen UK; ^2^ Programa de Pós‐graduação em Ecologia e Conservação Universidade Federal do Paraná Curitiba Brazil

**Keywords:** ageing, ecological specialisation, precision nutrition

## Abstract

Animals often regulate the intake and quantity of nutrients to maximise fitness through life‐history traits such as lifespan, but we still lack a proper understanding of how specific nutrients influence these traits. Here, I developed an algorithm which allowed me to create a nutrient‐specific database from literature data, and investigated how the requirements of protein (P) and carbohydrate (C) needed to maximise lifespan evolved across nine insect species. I found moderate evidence of a phylogenetic signal on the optimal ratio of protein to carbohydrate ratio (PC ratio) that maximised lifespan, suggesting that optimal PC ratio for lifespan could have evolved non‐independently among related species. I also found evidence for weak‐to‐strong sex‐specific optimal PC ratios for lifespan, suggesting that sex‐specific nutritional needs to maximise lifespan can emerge and persist in some species. Although limited in the number of species, the approach adopted here is portable to experiments with n number of nutrients and, thus, can be used in complex comparative precision nutrition studies for insights into the evolution of animal nutrition.

## Graphical abstract

Does one diet feed them all? Morimoto developed an algorithm to study whether the protein and carbohydrate ratios (PC ratios) that maximise lifespan are shared among related insect species. Related species likely share optimum PC ratios for lifespan. There is evidence of sex‐specific optimal PC ratios, indicating nutritional trade‐offs between males and females in the same species. Such sex‐specific trade‐off may also be shared among related species.

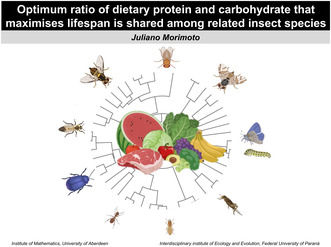

**Abbreviations**
GF: geometric Framework
PC ratio: protein‐to‐carbohydrate ratio

Different animals have different nutritional needs (Sauvant et al., 2008; Wu, 2017). This appears obvious at a coarse level since animals feed on different food types, but how nutritional needs differ among species at the nutrient level remains an open question. Nutrient‐specific needs are not always evident from the food type of a species. For instance, animals that seemingly feed on different food types can converge towards similar nutrient profiles, as is the case of the giant panda which, although classified as a strict herbivorous species, has evolved a protein‐biased diet akin to that of carnivores (Nie et al., 2019). Thus, it is important to look beyond food types and into nutrient‐specific patterns to gain insights into the evolution of nutritional needs across the tree of life (González et al., 2017; Machovsky‐Capuska et al., 2016).

Comparative nutrition has long been recognised as a powerful approach to uncover how nutritional needs have evolved (Baker & Czarnecki‐Maulden, 1991; Mitchell, 1962; Payne & Wheeler, 1968). It was used to provide insights into the effects of both macro‐ and micronutrients on health (see Baker & Czarnecki‐Maulden, 1991; Lei et al., 2022, references therein), including the effects of protein on morphological and reproductive traits (Swanson et al., 2016; Talal et al., 2023). Comparative nutrition studies are few because it is difficult to obtain a single dataset that maps the effects of specific nutrients to life‐history traits, such as lifespan or reproduction. A more parsimonious approach is to identify multiple but comparable studies on different species which allow for comparative studies. A recent study by Swanson et al. (2016) showed the effects of nitrogen, phosphorus and sodium across 96 butterfly species, and found that species feeding on high nitrogen diets are more fecund but have smaller eggs, potentially highlighting the evolution of a nutritional trade‐off. A limitation in this study is that dietary nitrogen may be used as proxy for protein intake and protein needs, but these are not necessarily equivalent (Mattson Jr, 1980). Nakagawa et al. (2012) used the comparative approach to study lifespan extension via dietary restriction across 36 species and showed the proportion of protein intake had greater influence on lifespan extension than the degree of caloric restriction. These studies highlight the need for studies using nutrient‐specific approaches.

The Geometric Framework for Nutrition (GF) is an experimental framework which allows for the study of nutrient‐specific effects on life‐history traits in animals and humans (Raubenheimer & Simpson, 1993; Simpson & Raubenheimer, 1993). GF is portable to any species, which makes GF an attractive framework for insights into the evolution of nutrition (Raubenheimer & Simpson, 2020; Simpson & Raubenheimer, 2012) as well as a potential key for precision nutrition experiments and its future applications (Simpson et al., 2017). GF has gained popularity and has underpinned major insights into animal and human nutritional ecology over the last decades, primarily from studies in insect species but also more recently in vertebrate models and humans (see e.g., Barragan‐Fonseca et al., 2019; Behmer, 2009; Bradbury et al., 2014; Lee et al., 2008; Maklakov et al., 2009; Ng et al., 2018; Pascacio‐Villafán et al., 2022; Polak et al., 2017; Ponton et al., 2015; Rapkin et al., 2018; Simpson et al., 2003, 2017; Simpson & Raubenheimer, 2005; Solon‐Biet et al., 2014, and references therein). Thus, GF datasets are unique in their potential to reveal broad‐scale patterns of nutrition, making GF the ideal candidate for large‐scale comparative nutrition studies. However, open data practices in the field remain poor, preventing broad‐scale comparative studies (Morimoto & Lihoreau, 2020). We therefore lack the means to study how optimum nutrient intakes evolved using a systematic and nutrient‐explicit comparative approach.

I conducted a systematic literature search to assess the availability of raw data in the GF literature (see Section 1), I found that out of 32 studies using the GF framework to study the ratio of protein (P) and carbohydrate (C) in the diet and its effects on lifespan in male and/or female insects, only five (29.4%) provided direct access to the raw data (see Figure 1a). This is in line with my previous report on the open practices in the field which showed that only ca. 39% of general (i.e. not limited to a particular trait) GF studies made their raw data available (Morimoto & Lihoreau, 2020). Among the identified studies, there was a notable sex‐bias: all (100%) studies investigated female lifespan in response to diet, while only eight studies (25%) also investigated male lifespan in response to diet.

**FIGURE 1:**
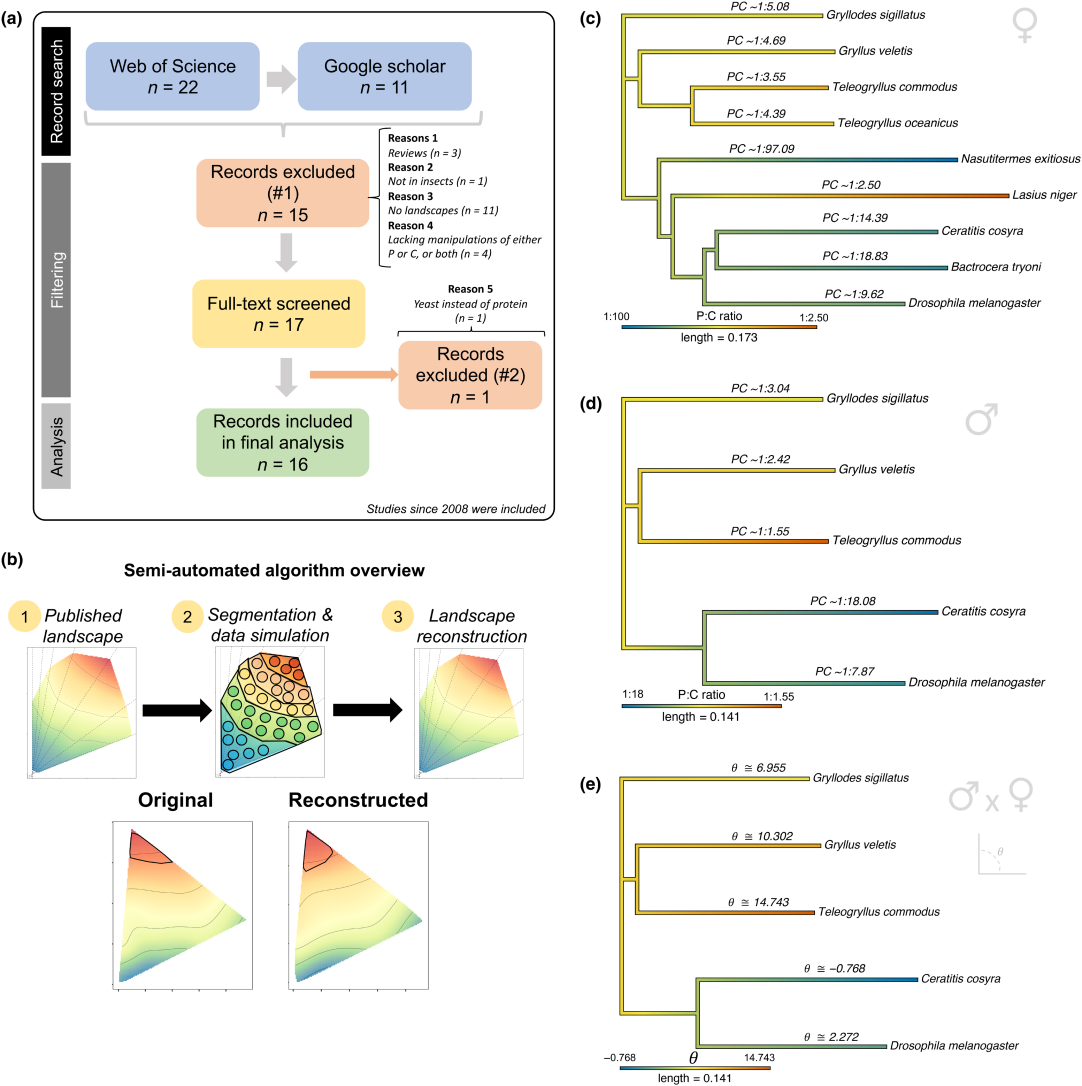
Optimum PC ratio across related insect species (a) Flowchart for the systematic literature search of experimental studies in insects that used GF to investigate the PC ratio effects on lifespan. (b) Overview of the semi‐automated algorithm to extract information of performance landscapes for lifespan from the published GF literature. (c) Phylogeny mapping the PC ratio that maximises lifespan across insect species for which GF data are available in the published literature (females). (d) Phylogeny mapping the PC ratio that maximises lifespan across insect species for which GF data are available in the published literature (males). (e) Phylogeny mapping the angle θ, which is a metric of nutritional trade‐off, for PC ratios that maximise lifespan across insect species in the database (note that the phylogeny is limited by the sex with the least amount of information, i.e. males). Negative values for the angle θ indicate that female optimal PC ratio was lower than the optimal PC ratio of males.

Open data practices are widely recognised as an imperative for research breakthroughs (Lowndes et al., 2017; Murray‐Rust, 2008; Reichman et al., 2011). Thus, innovative ways that open GF datasets for comparative studies are urgently needed to advance our understanding of animal nutrition. To overcome the lack of data availability, I developed a semi‐automated algorithm which enabled me to reconstruct published performance landscapes from GF studies that do not make their raw data available (see Figure 1b and ‘Experimental procedures’ section for details). While this does not replace the need for better open data practices, it allowed me to create the first systematic precision nutrition database for comparative nutrition insights (Morimoto & Lihoreau, 2020). For practical and financial reasons, GF has been most commonly used in insect studies and this study and database focused on this group, although it is portable to any animal group. The algorithm allowed me to create a database with optimal PC ratios for lifespan across nine insect species for females and five insect species for males.

I firstly tested the hypothesis that the optimal PC ratio that maximise lifespan is shared among related species, that is, optimal PC ratio is non‐independent among species with shared evolutionary history. I measured phylogenetic signal, which is a measure of non‐independent evolution of a continuous trait (in this case, optimal PC ratio) using two common metrics: (a) Pagel's λ, which is well‐defined within the range of 0 (no signal) and 1 (strong signal) and (b) Blomberg's K which varies between 0 and ∞ and is proportional to the strength of the phylogenetic signal, whereby K=1 indicates no difference between the trait evolution as expected under Brownian motion. These metrics measure different aspects of the phylogenetic signal, the former measuring a scaling parameter relative to expectation under Brownian motion and the former, the normalised ratio of variances within and between clades over the expected variances under Brownian motion (see Münkemüller et al., 2012; Revell et al., 2008; Revell & Harmon, 2022, for more details). Because of the female‐bias representation in the database, the mapping of the optimal PC ratio for lifespan onto the phylogeny was more extensive for females than males, and was done separately, giving less precise (i.e. higher variance) estimates of the posterior distribution of the phylogenetic signal for males. I used the Nutrigonometry model to the reconstructed performance landscapes to compute the optimal PC ratio region in the performance landscapes that maximised lifespan in each species (Morimoto et al., 2022, 2023; Morimoto & Lihoreau, 2019) (see Section 1 for details).

The results revealed a moderate phylogenetic signal for the PC ratio that maximised female lifespan (Blomberg's K: 1.265; *p* = 0.021; MCMCglmm Pagel's λ: 0.580, HPD interval: [0.222, 0.938]) and male lifespan (Blomberg's K: 1.228; *p* = 0.032; MCMCglmm Pagel's λ: 0.445, HPD interval: [0.021, 0.901]). In females, Orthopterans had extended lifespan when fed at intermediate PC ratios. Within holometabola, termites and Dipterans had extended lifespan in low PC ratios while the ant *L*. *niger* showed the opposite effect, with extended lifespan at high PC ratios (Figure 1c). In males, the patterns were similar despite the lack of Hymenopterans (see Figure 1d). These results must be interpreted with caution though, due to the relatively small number of species used in the comparative model to estimate phylogenetic signal. This led to relatively wide credible intervals for the estimates of Pagel's λ as well as a noticeable sensitivity to prior specifications on the estimates of Pagel's λ (see Dialog S1). With more data being generated in the field, these estimates will inevitably become less variable. Nonetheless, these results show for the first time that the optimal PC ratio for lifespan may be shared among related insect species.

Species that live longer in diets with higher or lower PC ratio may also feed on diets with similar PC ratios when given a choice. However, I found no evidence of a positive relationship between optimal PC ratio that maximise lifespan and self‐regulated PC ratios in both sexes in the database (*Females*: Mean posterior[95% CI]: 0.405 [−0.452, 1.323]; *Males*: −0.531 [−4.041, 4.024]). This does not imply that the relationship between optimal PC ratio and self‐regulated PC ratio is absent: multiple studies in single insect species have shown that, when given a choice, self‐regulated PC ratios often match more closely the optimal PC ratio for reproductive traits (see Hawkes et al., 2022; Jensen et al., 2015; Lee et al., 2008; Maklakov et al., 2008, and references therein). Hosking et al. (2019) showed through simulations that self‐regulated PC ratio that optimises reproduction should be prioritised under certain evolutionary contexts. Thus, it is possible that the relationship between optimal PC ratio and self‐regulated PC ratio exists for reproductive traits, but this remains to be tested.

Optimal PC ratios may also vary within species between sexes, which creates the potential for sexual conflict over optimal nutrition (Carey et al., 2022; Jensen et al., 2015). This can lead to long‐term ‘tug‐of‐war’ between sexes over nutrient intake or alternatively, lead to the evolution of physiological and behavioural adaptations to resolve—or mitigate—the negative effects of the conflict when both sexes cannot achieve their optimal nutrition (Hawkes et al., 2022; Simpson & Raubenheimer, 1993, 1999). Using the database, I tested whether there was evidence for a phylogenetic signal in the sex‐specific optimal PC ratio for lifespan in insects. I used the recently developed Nutrigonometry model to compare nutritional trade‐offs in the PC ratio (angle θ) and the quantity (the hypothenuse) of the diets that extended lifespan. There was evidence of moderate phylogenetic signal for the angle θ but not for the hypothenuse, captured by Blomberg's K but not Pagel's λ (θ: Blomberg's K: 1.264; *p* = 0.027; MCMCglmm Pagel's λ: 0.0564, HPD interval: [0.00, 0.548]; *Hypothenuse*: Blomberg's K: 0.900; *p* = 0.792; MCMCglmm Pagel's λ: 0.0002, HPD interval: [0.000, 0.001]). The nutritional trade‐off measured by θ appeared to be stronger in Orthopterans, particularly *T*. *commodus*, compared with Dipterans (see Figure 1e). These results show that sex‐specific nutritional needs are likely related to the nutrient balance rather than nutrient quantity.

Physiological and behavioural traits that regulate optimal PC ratio intakes are likely under selection. The findings that optimal PC ratio is shared among species also suggest that the underlying physiological machinery to cope with diet balances can also be shared. The database created here did not allow me to investigate shared molecular pathway activities underpinning the effects of PC ratios on lifespan. However, it is likely that the nutrient‐sensing mTOR pathway is involved on the evolution of optimal PC ratio intake on lifespan, considering taxa‐specific and comparative evidence for the role of this pathway in diet‐dependent lifespan extension (Garratt et al., 2016; Ma & Gladyshev, 2017). Furthermore, a recent comparative transcriptome study across 14 Drosophila species also suggests that lifespan regulation involves a complex orchestra of system‐level gene expression and trade‐offs (Ma et al., 2018), suggesting that physiological trade‐offs emerging from responses to diet may also be shared among closely related species. Future studies should test whether mTOR expression level is linked to the PC ratios that extend lifespan across species and whether mTOR expression—and the expression and trade‐offs across nutrient‐sensing pathways—evolves non‐independently along with PC ratios. Diet‐dependent comparative transcriptome analysis will shed light onto the pathways and trade‐offs that are conserved in the responses to diet. It is also worth mentioning that some traits are plastic and the location of peaks in GF landscapes might vary depending on environmental conditions such as temperature (Chakraborty et al., 2020; Kutz et al., 2019; Lee & Roh, 2010). This has been shown for developmental traits such as developmental time and growth rate but we do not know whether similar patterns are seen for lifespan. This remains subject of future studies.

Genetic constrains may prevent rapid evolution of dietary choice as well as the physiological machinery to resolve sex‐specific optimal diet intake. For example, Hawkes et al. (2022) showed that positive genetic correlations under diet choice can accelerate (males) or constrain (females) evolutionary responses to PC ratio intake in *G*. *sigillatus*. In *D*. *melanogaster*, both sexes display considerable heritability on the intake of both protein and carbohydrates (Reddiex et al., 2013). However, it is unknown how selection in response to diet acts between sexes across species, making it difficult to hypothesise the underlying factors driving the maintenance or resolution of sexual conflict over nutrition. A recent nutrient‐explicit model showed that selection acting on macronutrient appetite can modulate PC ratio intakes and influence the prioritisation of life‐history trait optimisation under different ecological conditions in an asexual population (Hosking et al., 2019). A generalisation of this model to include how intake targets evolve in sexual populations with sex‐specific nutritional needs for optimal expression of competing life‐history traits will provide important insights into the evolution and maintenance of sexual conflict over nutrition.

The main limitation of this study is the relatively low number of species for which GF lifespan landscapes are available. It was possible to retrieve GF landscapes only for nine species of insects for the female database and five insect species for the male database. With the low sample size, it is not surprising that the MCMC models to estimate phylogenetic signal displayed sensitivity to prior specifications (see Dialog S1). Nonetheless, in all cases, even non‐informative priors found on average a moderate estimate for the Pagel's λ as measure of phylogenetic signal (Dialog S1). Is in unquestionable that more studies, in different species, are urgently needed, as they will allow for more robust estimates of phylogenetic signals in the optimal PC ratio maximising lifespan and other life‐histories. It is also worth noting that the diet formulations of the studies identified here varied, with studies in Orthopterans relying on powdered diets derived from Simpson & Abisgold (1985) while studies for other groups using primarily gel‐based diets (but see Carey et al., 2022). The impacts of the diet formulation on physiological responses and lifespan were assumed to be negligible, but this remains a key factor to be tested empirically in the field.

In conclusion, I started off from the common knowledge that different animals eat different foods and asked the question: is there one ratio of protein and carbohydrate that maximises lifespan across species? Using insects as case study, I showed that there may exist such shared optimal PC ratio for lifespan among related species. This is likely to be true for other life‐histories, making the comparative approach proposed here an essential part of future precision nutrition predictive models. Although relatively modest in the number of species compared to other non‐GF comparative nutritional studies (Swanson et al., 2016), this study adopts a direct nutrient‐specific approach and lays the foundation for the use of GF nutritional data to gain insights into the evolution of nutritional needs across taxa.

## 1 EXPERIMENTAL PROCEDURES

Literature search in the Web of Science database was conducted to identify all studies since 2008 that used GF to study lifespan (Search terms: Geometric Framework, nutrition, lifespan OR survival [All fields]). The search was supplemented with new entries from Google Scholar search with same terms (Figure 1a). In total, 32 studies were identified from which 17 complied with the inclusion criteria, which were:

1. Presents experimental data;
2. Conducted in an insect species;
3. Displayed GF performance landscapes for lifespan;
4. Presents results for diet manipulations of protein and carbohydrate ratios (PC ratios).

Criteria number (2) was adopted because there is a general lack of controlled experimental studies using GF to assess lifespan in non‐insect groups (but see Solon‐Biet et al., 2014). Methods and data availability statements for each of the included studies were read, and raw data were downloaded when available for model validation. The female database included nine different species across three insect orders (Orthoptera, Hymenoptera, and Diptera). The male database included eight studies, from which five species and two orders (Orthoptera and Diptera) were represented. Two studies presented multiple landscapes for lifespan and a decision was made as to which landscape to include in the present study. For instance, Fanson et al. (2012) presented lifespan performance landscapes for mated and virgin females, while Poissonnier et al. (2018) presented two lifespan performance landscapes for worker and soldier termites. In these cases, I selected the performance landscapes for virgins and workers, respectively, as representative of these studies and species. One study in *D*. *melanogaster* was excluded from the analysis because the performance landscape for lifespan presented in the original study was given as yeast‐to‐sugar, rather than protein‐to‐carbohydrate ratio (Skorupa et al., 2008). The list of all studies used in this analysis is provided in Table 1.

**TABLE 1:** List of articles and associated species for which the performance landscape for lifespan was extracted using the semi‐automated algorithm developed in this study.

Species	Year	Source title	Authors
*Drosophila melanogaster*	2022	Biogerontology	Carey, MR; Archer, CR; Rapkin, J; Castledine, M; Jensen, K; House, CM; Hosken, DJ; Hunt, J
*Drosophila melanogaster*	2015	Aging Cell	Jensen, K; McClure, C; Priest, NK; Hunt, J
*Teleogryllus oceanicus*	2019	Journal of Evolutionary Biology	Ng, SH; Simpson, SJ; Simmons, LW
*Bactrocera tryoni*	2009	Aging Cell	Fanson, BG; Weldon, CW; Perez‐Staples, D; Simpson, SJ; Taylor, PW
*Ceratitis cosyra*	2017	Ecology and Evolution	Malod, K; Archer, CR; Hunt, J; Nicolson, SW; Weldon, CW
*Teleogryllus commodus*	2015	Antioxidants	Archer, CR; Hempenstall, S; Royle, NJ; Selman, C; Willis, S; Rapkin, J; Blount, JD; Hunt, J
*Bactrocera tryoni*	2012	Proceedings of the Royal Society B	Fanson, BG; Fanson, KV; Taylor, PW
*Bactrocera tryoni*	2012	Age	Fanson, BG; Taylor, PW
*Teleogryllus commodus*	2008	Current Biology	Maklakov, Alexei A., Stephen J. Simpson, Felix Zajitschek, Matthew D. Hall, Josephine Dessmann, Fiona Clissold, David Raubenheimer, Russell Bonduriansky, and Robert C. Brooks.
*Drosophila melanogaster*	2008	PNAS	Lee, Kwang Pum, Stephen J. Simpson, Fiona J. Clissold, Robert Brooks, J. William O. Ballard, Phil W. Taylor, Nazaneen Soran, and David Raubenheimer
*Drosophila melanogaster*	2008	Aging Cell^a^	Skorupa, Danielle A., Azra Dervisefendic, Jessica Zwiener, and Scott D. Pletcher
*Gryllus veletis*	2014	Proceedings of the Royal Society B	Harrison, Sarah J., David Raubenheimer, Stephen J. Simpson, Jean‐Guy J. Godin, and Susan M. Bertram
*Lasius niger*	2012	Proceedings of the Royal Society B	Dussutour, Audrey, and Stephen J. Simpson
*Gryllodes sigillatus*	2022	Functional Ecology	Hawkes, Michael, Sarah M. Lane, James Rapkin, Kim Jensen, Clarissa M. House, Scott K. Sakaluk, and John Hunt
*Teleogryllus commodus*	2017	Evolution	Rapkin, J; Archer, CR; Grant, CE; Jensen, K; House, CM; Wilson, AJ; Hunt, J
*Drosophila melanogaster*	2018	Entomologia Experimentalis et Applicata	Semaniuk, Uliana, Khrystyna Feden'ko, Ihor S. Yurkevych, Kenneth B. Storey, Stephen J. Simpson, and Oleh Lushchak
*Nasutitermes exitiosus*	2018	Functional Ecology	Poissonnier, L. A., Arganda, S., Simpson, S. J., Dussutour, A., & Buhl, J.

^a^ Study excluded from the analysis because performance landscapes were drawn using yeast: sugar ratios rather than protein: carbohydrate ratios.

Data extraction and analyses were conducted in R version 4.1.3 (R Core Team, 2022) (see Figure 1b). The semi‐automated algorithm to extract simulated data to reconstruct the published lifespan performance landscapes worked as following:

1. The algorithm segmented the performance landscapes with respect to the *z*‐axis using the ‘recolorize’ 0.1.0 package (Weller et al., 2022). In this study, the *z*‐axis corresponds to lifespan values.
2. Each z‐layer was manually assigned a value for lifespan based on the contour lines depicted in the image on the published literature. This ensured that the values and ranges of lifespan matched that of the original landscape.
3. This approach ‘categorises’ the values for lifespan, leading to a *z*‐axis that was ladder‐like. This format hinders the estimates of the performance landscape using thin‐plate splines from the ‘fields’ package (Nychka & Nychka, 2003). To overcome this, I added random error to the categorised lifespan values by sampling from a normal distribution using the ‘rnorm’ function, with mean 0 and standard deviation 0.25. Note that, without the raw data, it is currently impossible to estimate the true variance underlying the data used to build performance landscapes (see comment below).
4. A filtering function was applied to remove noise introduced during the segmentation step which could lead to simulated points existing outside the true region of the original performance landscape.
5. The reconstructed landscapes were drawn using a custom‐made function which is a wrapper for the ‘Tps’ function from the ‘fields’ package (Nychka & Nychka, 2003).

Note that without the raw data, it is virtually impossible to estimate the variance in the *z*‐variable. By simulating the standard error from a normal distribution with mean 0 and standard deviation 0.25 throughout, I assumed that variance in lifespan across species and diets with varying PC ratios were constant. This might not reflect reality, but it is nonetheless an essential assumption for data interpolation and identification of optimal PC ratios in reconstructed performance landscapes. Future studies to reconstruct the true variance from published images are needed. Landscapes were plotted using the ‘ggplot2’ 3.4.2 package (Wickham, 2016). Data handling was conducted using the tidyverse packages ‘dplyr’ 1.1.2 and ‘tidyr’ 1.2.0 (Wickham et al., 2019).

Raw data for seven studies were available and allowed me to validate the algorithm above. Validation was done using the Nutrigonometry model to the reconstructed and original lifespan performance landscapes and measuring whether the angle θ and the hypothenuse of the identified peak region differed between the original and reconstructed landscapes. Average estimates along with 95% confidence intervals for the estimates were calculated from the model. Zero‐overlapping estimates, which were found for all seven studies, showed that the reconstructed landscapes displayed its peak region in a comparable location to the landscapes made using the original data (see Figures S1 and S2). Validation was done using the female database due to accessibility to a higher number of raw data for female landscapes. Given the same methodology was implemented to extract and reconstruct both male and female landscapes, and male and female landscapes do not vary dramatically, this validation approach is suitable. Reconstructed landscapes and their estimated peak regions used in this study are given in Figure S3.

Phylogenetic relationship between the species in the database was reconstructed using the whole or partial mitochondrial genome (i.e. the barcoding CO1 gene sequence) obtained from the NCBI database, using the packages ‘ape’ 5.6‐2 (Paradis & Schliep, 2019), ‘phytools’ 1.2.0 (Revell, 2012) and ‘phyloseq’ 1.38.0 (McMurdie & Holmes, 2013). Multiple sequence alignment was done using the ‘msa’ 1.26.0 package (Bodenhofer et al., 2015). Blomberg's K and its statistical significance were estimated using the ‘phylosig’ function in the ‘phytools’ package; phylogenetic tree with continuous trait mapping were made using the ‘contMap’ function from the same package. Pagel's λ was estimated using the ‘MCMCglmm’ package and the function with same name (Hadfield, 2010). Because of the unequal number of species, phylogenetic signals were estimated for each sex separately.

MCMCglmm models with gaussian family error distribution were fitted using optimal PC ratio as response variable with a model with fixed intercept and study and species as random variables, accounting for the non‐independent variance–covariance matrix from the reconstructed phylogenetic relationships among species. Fixed effect prior parameters were set as *V* = 1 and nu = 1 while random effect priors were set at *V* = 1 and nu = 0.02. Burn‐in was set at 2000, thin parameter at 20 and a total of 20,000 iterations for all models. Convergence was analysed using autocorrelation for fixed and random effects as well as Gelman‐Rubin diagnostics for among‐chain convergence. Prior sensitivity tests were conducted for optimal PC ratio models using a range of values for the parameter nu (i.e. $10^{-6}$, 0.002, 0.02 and 0.2) (see Dialog S1). Sex‐specific optimal PC ratio where compared using the angle θ and the hypothenuse obtained using the Nutrigonometry model (Morimoto et al., 2023). θ and the hypothenuse were calculated within each study and species and the averages of these estimates per species were used for the analysis of phylogenetic signal as described above. Phylogenetic signal for sex‐specific optimal PC ratio could only be calculated for the species in which both male and female data were available and therefore was limited to only a subset of the female database (see above). The MCMCglmm models to estimate Pagel's λ had either the angle θ or the hypothenuse as response variable and a fixed intercept, with study and species as random effects as described above. I used default MCMCglmm priors for these models. The relationship between optimal PC ratio for lifespan and self‐regulated PC ratio was analysed using a MCMCglmm model with optimal PC ratio as response variable and self‐regulated PC ratio as independent variable, controlling for the random effects of study, species and observation (to account for repeated measures in the same study and species). I used the same values of V and nu for priors as in the above models with the addition of priors for the random effect of observation with parameters V=1 and nu=0.002. The highest posterior density intervals were calculated using the ‘HPDposterior’ function of the ‘MCMCglmm’ package for all MCMC models.

## AUTHOR CONTRIBUTIONS

JM is the sole author of this manuscript and was responsible for conceptualising the model, coding, data analysis, manuscript preparation and submission.

## FUNDING INFORMATION

JM is supported by the Biotechnology and Biological Sciences Research Council [BBSRC: BB/V015249/1].

## CONFLICT OF INTEREST STATEMENT

None declared.

## Supporting information


Data S1.

Figure S1.–S3.

## Data Availability

Data on optimal PC ratio that maximise lifespan are available as Supporting Information. Code for the semi‐automated algorithm is not made available because the author, who developed the algorithm, is still using the algorithm to create and analyse other databases with similar approach to that described here.
