# Supplementary material for: Optimum ratio of dietary protein and carbohydrate that maximises lifespan is shared among related insect species
Source: Aging Cell. 2023 Dec 13;23(3):e14067. doi: 10.1111/acel.14067 (PMC10928577; doi:10.1111/acel.14067)
Supplement: Supplementary file 1 — Data S1. [file ACEL-23-e14067-s001.zip › Dialog S1.docx]

**Dialog S1. MCMCglmm models.**

MCMCglmm models were used to estimate phylogenetic signal via Pagel’s lambda. I conducted a prior sensitivity analysis where I ran models with priors of varying levels of confidence (i.e. *V* = 1, *nu* ranging from 0.2 to 10^-6^; see main text). These priors expanded on those tested in Nakagawa et al., (2012). Results suggest that estimates of Pagel’s lambda were somewhat sensitive to prior specifications for both sexes (but more so for males which had smaller number of species), although the posterior mean for all models, irrespective of priors, found a moderate strength of phylogenetic signal (with wide credible interval estimates; see also main text for Blomberg’s *K*). Because of my *a priori* belief, based on the plots of the optimal PC ratio onto the phylogeny during the exploratory data analysis stage, I selected model 2 (*V* = 1, *nu* = 0.02), which was a resonable balance between uninformative prior and a prior with more confidence, when presenting the results, while adding a cautionary tale for the interpretations of the results given prior sensitivity (see Main text). Convergence of the final models were analysed using autocorrelation for fixed and random effects (which were close to or lower than 0.1) as well as Gelman-Rubin diagnostics for among-chain convergence, whereby I ran the MCMCglmm with same specifications three independent times and compared the Gelman-Rubin score, which were 1.01 (upr: 1.02 and 1.01) for both male and female models, respectively. MCMCglmm models for theta and the hypothenuse, which used default priors, also converged (Gelman-Rubin: 1.01; upr: 1.01-1.02, Theta autocorrelation: intercept = 0.015, study = 0.02, species 0.00, units = 0.016; Hypothenuse autocorrelation: intercept = 0.03, study = 0.17, species 0.05, units = 0.004). Similarly, MCMCglmm models for the relationship between optimal and self-regulated PC ratios converged (Gelman-Rubin: 1.06; upr: 1.06, autocorrelation intercept = 0.019, self-regulated PC ratio: 0.021, study = 0.003, species = 0.051, units = 0.037).

**Table S1.** Prior sensitivity analysis for the phylogenetic signal on optimal PC ratio.

| ***Sex*** | ***model*** | ***V*** | ***nu*** | ***sample size*** | ***Pagel's lambda*** | ***lwr*** | ***upr*** |
| --- | --- | --- | --- | --- | --- | --- | --- |
|  | 1 | 1 | 0.002 | 900 | 0.332 | 0.0135 | 0.766 |
| **Females** | 2 | 1 | 0.02 | 900 | 0.58 | 0.2200 | 0.938 |
|  | 3 | 1 | 0.2 | 900 | 0.808 | 0.5650 | 0.977 |
|  | 4 | 1 | 10^-6 | 900 | 0.377 | 0.0000 | 0.888 |
|  | 1 | 1 | 0.002 | 900 | 0.35 | 0.0000 | 0.899 |
| **Males** | 2 | 1 | 0.02 | 900 | 0.445 | 0.0210 | 0.901 |
|  | 3 | 1 | 0.2 | 900 | 0.666 | 0.2680 | 0.992 |
|  | 4 | 1 | 10^-6 | 900 | 0.18 | 0.0000 | 0.858 |

**References**

Nakagawa, S., Lagisz, M., Hector, K.L. and Spencer, H.G. (2012), Comparative and meta-analytic insights into life extension via dietary restriction. Aging Cell, 11: 401-409. <https://doi.org/10.1111/j.1474-9726.2012.00798.x>
