# Supplementary material for: Optimum ratio of dietary protein and carbohydrate that maximises lifespan is shared among related insect species
Source: Aging Cell. 2023 Dec 13;23(3):e14067. doi: 10.1111/acel.14067 (PMC10928577; doi:10.1111/acel.14067)
Supplement: Supplementary file 2 — Figure S1.–S3. [file ACEL-23-e14067-s002.docx]

**Supplementary Figures**

**Figure S1.** Estimates of error between the peak regions of original (raw data) and reconstructed performance landscapes for lifespan. Estimates of peak region were done using the Nutrigonometry model (Morimoto et al., 2023 *Am Nat*). Note that the credible intervals are relatively wider for the hypothenuse in the Lee et al., (2008) and Harrison et al., (2014) because of the quantity of nutrients eaten by the experimental animals in these studies were higher than the others. Importantly, the hypothenuse measures trade-offs over quantity and, therefore, has to be calculated in the same scale as the data was collected (i.e. cannot be standardised).

**Figure S2.** Original and reconstructed landscapes. Red polygon represents estimated peak region (Nutrigonometry) (Morimoto et al., 2023 *Am Nat*).

**Figure S3.** Reconstructed landscapes used in this study. Red polygon represents estimated peak region (Nutrigonometry) (Morimoto et al., 2023 *Am Nat*).
